# A transposon insertion in *CmKNAT2-like2* disrupts mottled rind formation in melon (*Cucumis melo* L.)

**DOI:** 10.1093/hr/uhaf195

**Published:** 2025-07-28

**Authors:** Shuai Li, Jing Feng, Xinxiu Chen, Yuanchao Xu, Yuhao Song, Fanfan Chen, Yang Li, Naonao Wang, Jianlei Sun, Zhonghua Zhang, Sen Chai

**Affiliations:** College of Horticulture, Qingdao Agricultural University, Qingdao 266109, China; Shandong Key Laboratory of Bulk Open-field Vegetable Breeding, Ministry of Agriculture and Rural Affairs Key Laboratory of Huang Huai Protected Horticulture Engineering, Institute of Vegetables, Shandong Academy of Agricultural Sciences, Jinan 250100, China; Genome Analysis Laboratory of the Ministry of Agriculture, Agricultural Genomics Institute at Shenzhen, Chinese Academy of Agricultural Sciences, Shenzhen 518124, China; College of Horticulture, Qingdao Agricultural University, Qingdao 266109, China; College of Horticulture, Qingdao Agricultural University, Qingdao 266109, China; Genome Analysis Laboratory of the Ministry of Agriculture, Agricultural Genomics Institute at Shenzhen, Chinese Academy of Agricultural Sciences, Shenzhen 518124, China; College of Horticulture, Qingdao Agricultural University, Qingdao 266109, China; College of Horticulture, Qingdao Agricultural University, Qingdao 266109, China; College of Horticulture, Qingdao Agricultural University, Qingdao 266109, China; College of Horticulture, Qingdao Agricultural University, Qingdao 266109, China; Shandong Key Laboratory of Bulk Open-field Vegetable Breeding, Ministry of Agriculture and Rural Affairs Key Laboratory of Huang Huai Protected Horticulture Engineering, Institute of Vegetables, Shandong Academy of Agricultural Sciences, Jinan 250100, China; College of Horticulture, Qingdao Agricultural University, Qingdao 266109, China; College of Horticulture, Qingdao Agricultural University, Qingdao 266109, China

## Abstract

The mottled rind is an important fruit external appearance trait that influences consumer preferences. Previous studies reported that *CmMt1* and *CmMt2* regulate rind mottling in melon, yet *CmMt2* has not been cloned. In this study, we developed near-isogenic lines (NILs) using the nonmottled rind ‘*13C*’ as the recurrent parent and mottled ‘*P114*’ as the donor parent, and screened a mottled rind mutant ‘*S249*’ by ethyl methanesulfonate mutagenesis of ‘*13C*’. Combined with these genetic materials, *CmMt2* was delimited to a 44-kb region on chromosome 2. Within this genetic interval, a CACTA-type TIR transposon insertion was detected in all nonmottled rind lines, and this insertion may lead to impaired nuclear localization and dimerization capability of *CmKNAT2-like2* encoding a homeobox protein through the loss of conserved ELK and Homeodomain. Further, CRISPR/Cas9-mediated knockout of *CmKNAT2-like2* confirmed its pivotal role in regulating mottled rind phenotype. In addition, transcriptome analysis suggested that the transposon insertion in *CmKNAT2-like2* results in nonmottled rind by disrupting chloroplast development and altering the expression of chlorophyll biosynthesis-related genes, and population analysis revealed that the transposon associated with *CmKNAT2-like2* has undergone selection in cultivated melons. Collectively, these results demonstrate that *CmKNAT2-like2* is the causal gene underlying *CmMt2*, which regulates mottled rind in melon.

## Introduction

Creatures have evolved a dizzying array of patterns: stripes, spots, diamonds, and even mazelike designs. For the creatures bearing them, these patterns play a vital role in survival adaptation and social communication. For humanity, these patterns lend species unique aesthetic and commercial value. The external appearance is an important indicator of fruit quality that significantly influences consumer preferences. Plant patterns are primarily formed by differential pigment deposition, including anthocyanins, carotenoids, and chlorophyll [1, 2]. In recent years, numerous key regulatory genes controlling fruit external appearance have been successfully cloned in various horticultural crops. Notable examples include the *VvmybA1* regulating skin color in grapes [3], the *CCD4b* influencing the red coloration of peel in citrus [4], and the *SlGLK2* associated with uniform light green fruit in tomato [5]. However, the key genes and their regulatory networks controlling striping patterns in horticultural crops remain poorly characterized.

Transposable elements (TEs) are the most ubiquitous components of genomes and serve as driving forces for species evolution. As early as 1948, Barbara McClintock first observed that transposons influenced the variegated pigmentation of maize kernels and leaves, uncovering their essential function in shapingphenotypic differences. TE insertions can lead to epigenetic modification, alter gene structure, and rewrite gene expression networks [6–9]. Extensive studies have demonstrated that structural variations have played crucial roles in specific agronomic traits, including fruit flavor, fruit color, and rind pattern, while most of these variations originate from TEs [10–15].

Melon (*Cucumis melo* L.) is an economically important vegetable crop that is cultivated and consumed worldwide. Recent advances in genomic research have enabled the identification and characterization of key genes regulating important agronomic traits, leading to significant improvements in fruit flavor and pathogen resistance. Evolving consumer preferences have elevated fruit external appearance as a priority trait in modern melon breeding programs. The mottled rind is one of the most distinctive phenotypic traits in melon. Previous genetic studies identified multiple loci controlling presence or absence of mottled rind, including *Mt* (*mottled rind*), *Mt-2* (*mottled rind-2*), *mt-2* (*spots on the rind*), *CmSp-1* (*C. melo spot-1*), *st* (*striped epicarp*), *st3* (*striped rind 3*), and *spk* (*speckled fruit epidermis*) [16–20]. Recent genome-wide association study (GWAS) detected significant signals on chromosomes 2 and 4 associated with mottled rind trait in melon [21]. Additionally, epistatic interactions were observed between two regulatory loci, *CmMt1* and *CmMt2*, with evidence suggesting that the rind color gene *CmAPRR2* may coincide with *CmMt1* [22]. A recent study employing forward genetics identified *CmAPRR2* as the candidate gene for the rind pattern in ‘shooting star’ accession, where the excision of a hAT-like transposon within this gene drives trait formation [23]. Two independent studies using recombinant inbred line populations mapped the *Mt2* locus to the terminal region of chromosome 2 [19, 24]. However, the *Mt2* locus has not yet been cloned, and the molecular mechanism underlying its regulation of mottled rind formation remains unclear.

Near-isogenic lines (NILs) differing in rind pattern were developed using nonmottled ‘*13C*’, mottled ‘*P114*’, and an ethyl methanesulfonate (EMS)-induced mottled mutant ‘*S249*’ from ‘*13C*’. Using these resources, the *Mt2* locus was mapped to a 44-kb region on chromosome 2. A TIR-type transposon inserted in the intron of CmKNAT2-like2 disrupted its structure in ‘*13C*’. The expression pattern and the Cas9 knockout mutant confirmed that *CmKNAT2-like2* is a key regulator of rind mottling. The TE insertion in *CmKNAT2-like2* affected its nuclear localization and dimerization, and impacted the expression of chloroplast-related genes and chloroplast development. Population analysis showed that the transposon insertion is widespread in natural melon populations and strongly correlates with melon mottled rind. This study elucidates a TE-driven molecular mechanism for rind mottling, offering both a genetic marker for breeding and insights into how transposons shape phenotypic diversity in cucurbits.

## Results

### *Mt2* was delimited to a 275-kb region with NIL-derived population

**Figure 1:**
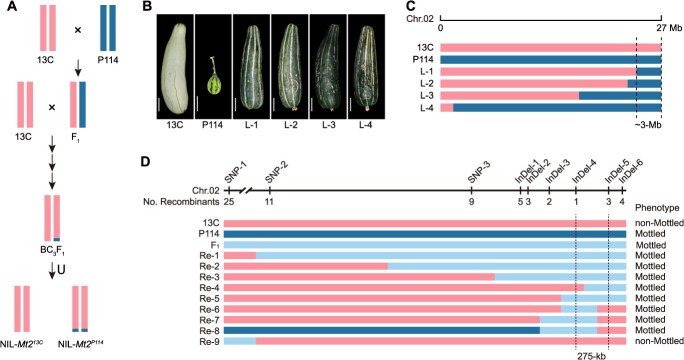
Genetic mapping of *Mt2* with NIL-derived population. (A) Schematic diagram summarizing the production of NILs with mottled and nonmottled rind. (B) Representative fruit images of NILs with the mottled rind. Bars, 5 cm. (C) Genotype of NILs bears mottled rind on chromosome 2. (D) *Mt2* was preliminary mapped to the 275-kb region with NIL-derived population.

Using the cultivated germplasm ‘*13C*’ with nonmottled rind as the recurrent parent and the wild germplasm ‘*P114*’ with mottled rind as the donor parent, NILs with the presence or absence of mottled rind were developed through crossing, backcrossing, and self-pollination (Fig. 1A). Genotype analysis revealed that four lines exhibiting mottled rind shared a 3-Mb segment at the end of chromosome 2, which is adjacent to the previously reported *Mt2* locus (Fig. 1B and C). This finding indicated that the mottled rind in ‘*P114*’ is regulated by *Mt2*, initially confining it to this 3-Mb region. To further refine the *Mt2* locus, line ‘L-1’, which carries the smallest fragment, was crossed with the ‘*13C*’ to construct an F_2_ segregating population. Single nucleotide polymorphism (SNP) and insertion–deletion (InDel) variations between parental lines were converted into molecular markers for genotyping the 270 F_2_ individuals. By integrating genotypic and phenotypic data from recombinant individuals, the *Mt2* locus was precisely mapped to a 275-kb region between InDel-4 and InDel-5 (Fig. 1D).

### *Mt2* was delimited to a 300-kb region with ethyl methanesulfonate-induced mutant-derived population

**Figure 2:**
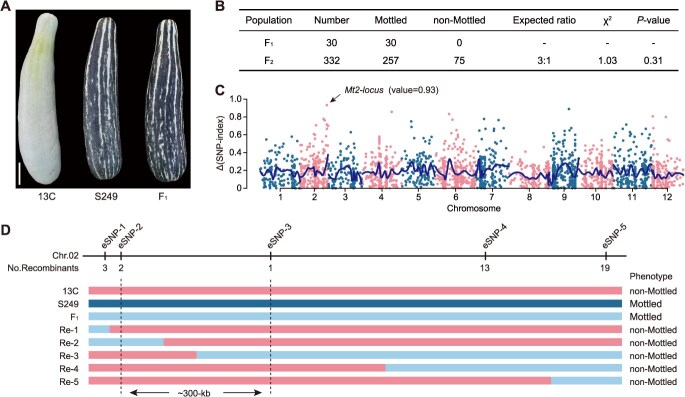
Genetic mapping of *Mt2* with ‘*S249*’-derived population. (A) Rind phenotype of ‘*13C*’, mottled mutant ‘*S249*’, and the hybrid F_1_. Bar, 5 cm. (B) Inheritance analysis of the mottled rind in ‘*S249*’. (C) MutMap^+^ identifies the candidate region responsible for the mottled rind phenotype in ‘*S249*’. (D) Preliminary mapping of *Mt2* locus with EMS-induced mutant-derived population.

The nonmottled germplasm ‘*13C*’ was previously subjected to mutagenesis using EMS to establish a mutant library. From this library, a mutant exhibiting a mottled rind, designated ‘*S249*’, was isolated. To elucidate the genetic inheritance of the mottled rind phenotype, a cross was made between ‘*S249*’ and ‘*13C*’. The F_1_ generation uniformly displayed the mottled rind trait, suggesting that mottling is a dominant characteristic over the nonmottled phenotype (Fig. 2A). Upon self-pollination of the F_1_ plants, an F_2_ population was produced. Phenotypic assessment and statistical evaluation of 332 F_2_ individuals showed that 257 plants bore mottled rinds, whereas 75 plants retained the nonmottled appearance. The observed mottled to nonmottled ratio adhered to the anticipated 3:1 Mendelian distribution (*χ*^2^ = 1.03, *P* = .31), implying that a single gene governs the mottled rind phenotype in the ‘*S249*’ mutant (Fig. 2B).

Subsequently, genomic DNA was isolated from 20 mottled and 20 nonmottled F_2_ plants to create M-pool and N-pool, which were then analyzed through whole-genome resequencing. MutMap^+^ analysis revealed that a pronounced genetic signal associated with rind mottling was identified at the terminus of chromosome 2, coinciding with the known *Mt2* locus (Fig. 2C). This finding indicated that the mottled phenotype in ‘*S249*’ is also influenced by the *Mt2* gene. Specific SNPs were then transformed into molecular markers based on the detected variants, and through fine mapping, the *Mt2* locus was confined to a 300-kb segment at the end of chromosome 2 between eSNP-2 and eSNP-3 (Fig. 2D).

### *CmKNAT2-like2* regulates the mottled rind formation in melon

**Figure 3:**
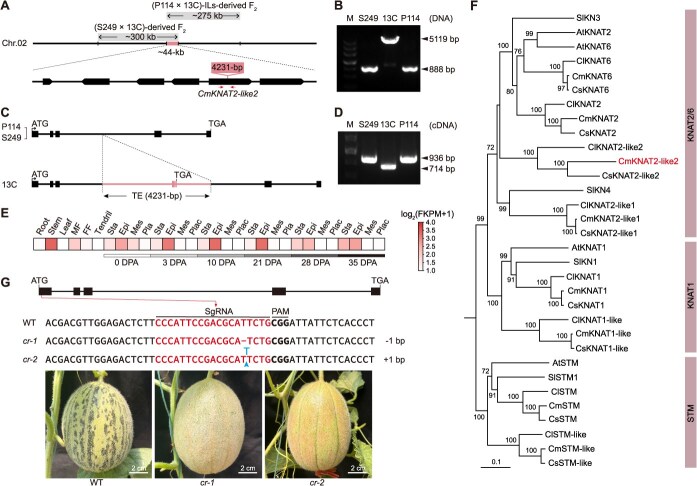
*CmKNAT2-like2* regulates the mottled rind formation in melon. (A) Fine mapping of *CmMt2* locus. Primers used for transposon insertion confirming are marked with arrows. (B) PCR validation of 4231-bp transposon in various parental lines. (C) Insertion of transposon altered the candidate gene structure in ‘*13C*’. (D) PCR validation of ‘*13C*’ and mutant transcript of candidate gene. (E) Spatial and temporal expression pattern of the candidate gene. (F) A phylogenetic tree showing relationships between the KNOX1 TFs (class I) from *Cucumis sativus* L., *C. melo* L., *Citrullus lanatus* L., *Solanum lycopersicum* L., and *Arabidopsis thaliana*. (G) CRISPR/Cas9-mediated knockout of *CmKNAT2-like2* resulted in nonmottled rind in ‘*P147*’. Bars, 2 cm.

The 275-kb interval identified from the NIL-derived population and the 300-kb interval pinpointed using the dominant mutant ‘*S249*’ overlap in a 44-kb region, encompassing six coding genes (Fig. 3A and Table S2). Compared to ‘*P114*’ and ‘*S249*’ with mottled rind, genomic sequence comparison uncovered a 4231-bp insertion within this interval in ‘*13C*’ (Fig. 3A). This structural variation was identified as a CACTA-type TIR transposon and was further confirmed using specific primers (Fig. 3B). In ‘*13C*’, the insertion of this transposon into the third intron of *MELO3C026289* introduces additional exons and a novel stop codon, thereby modifying its gene structure (Fig. 3C). RNA extracted from ‘*13C*’, ‘*S249*’, and ‘*P114*’ was reverse transcribed and amplified using primers designed based on the predicted gene structure. Sanger sequencing verified that the transcripts of *MELO3C026289^P114^* and *MELO3C026289^S249^* are 936 bp in length, whereas *MELO3C026289^13C^* produces a shorter transcript of 714 bp due to the transposon insertion (Fig. 3D). Expression pattern analysis revealed that *MELO3C026289* is expressed in the epicarp of fruit, consistent with the location of the mottled phenotype (Fig. 3E). Phylogenetic analysis revealed that *MELO3C026289* belongs to the KNAT2/6 subfamily, though it does not represent the most closely related member within this subfamily, and was therefore designated as *CmKNAT2-like2* (Fig. 3F). To further demonstrate the function of *CmKNAT2-like2* in mottled rind formation, we generated a construct targeting *CmKNAT2-like2* for CRISPR/Cas9-mediated genome editing. This construct was introduced into the melon inbred line ‘*P147*’, which has a mottled rind, is amenable to genetic transformation, and does not contain the TE insertion. Two null *CmKNAT2-like2* alleles, designated *cr-1* and *cr-2* with a 1-bp deletion and a 1-bp insertion, respectively, were obtained. Both alleles exhibited the expected nonmottled rind phenotype (Fig. 3G). These results indicated that *CmKNAT2-like2* is the causal gene underlying the *Mt2* locus, and the 4231-bp transposon is the causative variation responsible for absence or presence of mottled rind in melon.

### The transposon insertion disrupts the nuclear localization and dimerization capabilities of *CmKNAT2-like2* protein

**Figure 4:**
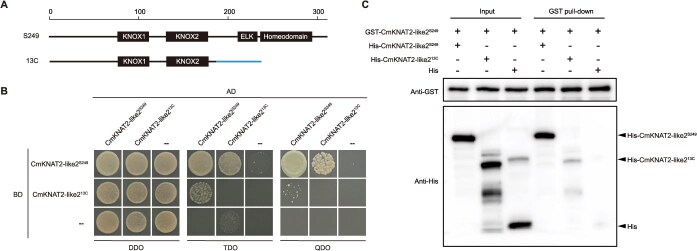
Detection of dimerization capability changes in CmKNAT2-like2 via Y2H and pull-down assays. (A) Conserved domain analysis of wild-type and mutant CmKNAT2-like2. (B and C) Assessment of dimerization capacity of wild-type and mutant CmKNAT2-like2 with Y2H (B) and pull-down assay (C).

Protein sequence analysis revealed that the wild-type CmKNAT2-like2*^S249^* contains four conserved domains: KNOX1, KNOX2, ELK, and Homeodomain. In contrast, the transposon insertion in CmKNAT2-like2*^13C^* results in the loss of the ELK and Homeodomain in C-terminal (Fig. 4A). Functional analysis of these domains indicates that KNOX1 is associated with transcriptional repression activity, KNOX2 is critical for dimer formation, ELK facilitates nuclear localization, and Homeodomain mediates DNA binding. To explore the functional implications of these structural alterations, we first generated GFP fusion constructs of CmKNAT2-like2*^S249^* and CmKNAT2-like2*^13C^* and examined their subcellular localization in tobacco leaves. Confocal microscopy revealed that GFP-CmKNAT2-like2*^S249^* localized predominantly to the nucleus, whereas GFP-CmKNAT2-like2*^13C^* exhibited diffuse fluorescence with a radiating pattern from the nucleus, suggesting impaired nuclear targeting (Fig. S1). Furthermore, we investigated the dimerization capacity of the two variants. Both yeast two-hybrid and pull-down assays consistently showed that CmKNAT2-like2*^13C^* had significantly diminished dimerization ability compared to CmKNAT2-like2*^S249^* (Fig. 4B and C). Taken together, these results demonstrate that the transposon insertion in CmKNAT2-like2*^13C^* disrupts its nuclear localization and dimerization capabilities, thereby severely impairing its function as a transcription factor.

### *CmKNAT2-like2* regulates chloroplast development and chloroplast-related gene expression

**Figure 5:**
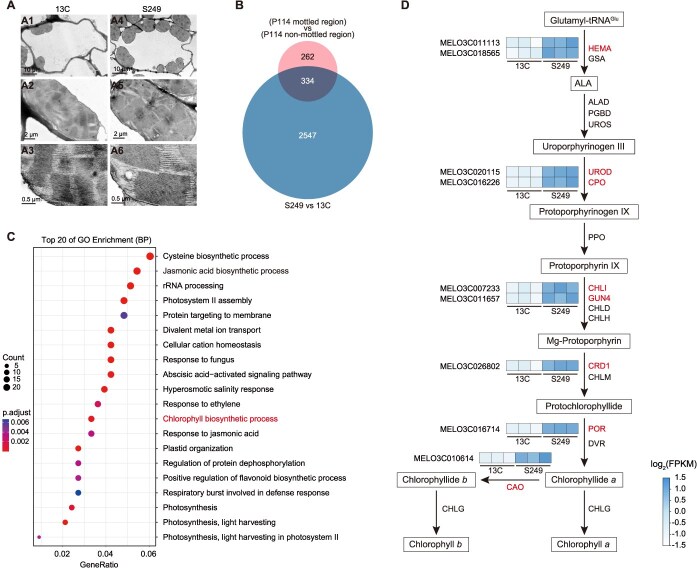
Transcriptome analysis of mottled and nonmottled regions in melon. (A) Transmission electron microscopy observation of chloroplasts in ‘*13C*’ (A1–A3) and ‘*S249*’ (A4–A6) fruit rind. (B) Venn diagram showing the DEGs between mottled and nonmottled region of ‘*P114*’ and DEGs between ‘*13C*’ and ‘*S249*’, respectively. (C) Top 20 biological process enrichment of shared genes between two groups of DEGs. (D) Simplified diagram of the chlorophyll biosynthetic pathway with heatmap of related DEGs between ‘*13C*’ and ‘*S249*’ marked.

Transmission electron microscopy analysis revealed a significantly higher number of chloroplasts and tightly stacking lamella structure of chloroplast in the epidermal cells of ‘*S249*’ compared to ‘*13C*’ (Fig. 5A). Observation of ‘*13C*’ and ‘*S249*’ fruits revealed that mottled rind initiates at early developmental stages (Fig. S2). To explore the possible molecular mechanism responsible for mottled rind formation, RNA-seq was performed with two sets of samples from ‘*P114*’, ‘*S249*’, and ‘*13C*’, respectively. The first set of samples consisted of the dark green mottled regions and the light green nonmottled regions of ‘*P114*’, while the second set of samples included the dark green mottled regions of ‘*S249*’ and the uniform light green rind of ‘*13C*’. The two sets of groups exhibited 596 (Table S4) and 2881 (Table S3) differentially expressed genes (DEGs), respectively, with 334 DEGs showing common expression patterns between both groups (Fig. 5B). Gene ontology (GO) pathway analysis of these 334 common DEGs revealed significant enrichment in terms related to photosynthesis and the chlorophyll biosynthesis process (Fig. 5C). Nine genes involved in the chlorophyll biosynthesis process were expressed differentially between ‘*13C*’ and ‘*S249*’ (Fig. 5D), including *glutamyl-tRNA reductase* (*HEME*), *uroporphyrinogen III decarboxylase* (*UROD*), *coproporphyrinogen III oxidase* (*CPO*), *Mg-chelatase I subunit* (*CHLI*), *regulator of Mg-chelatase* (*GUN4*), *Mg-proto IX monomethylester cyclase* (*CRD1*), *protochlorophyllide oxidoreductase* (*POR*), and *chlorophyllide a oxygenase* (*CAO*). Collectively, these results demonstrated that the mottled part of rind is due to an increased number of chloroplasts and elevated expression of chlorophyll biosynthesis process-related genes.

### Population analysis of the transposon element variant in *CmKNAT2-like2*

**Figure 6:**
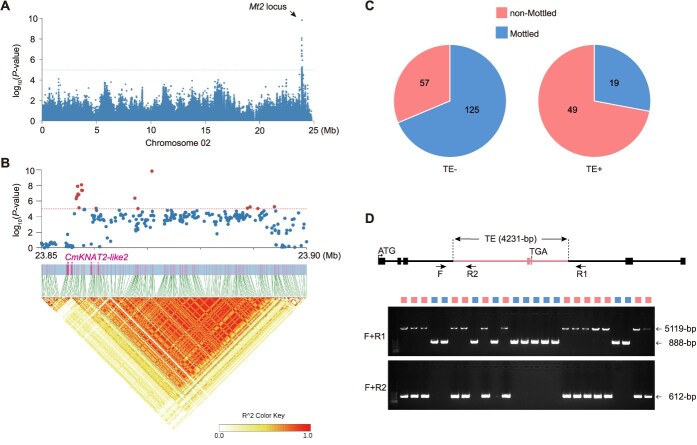
Population analysis of the transposon element variant in *CmKNAT2-like2*. (A) GWAS analysis of fruit rind mottling in melon (chromosome 2). (B) Local Manhattan plot (top) and LD plot (bottom) surrounding *Mt2*. (C) Correlation analysis of TE genotype and mottled rind phenotype across 250 accessions. (D) PCR validation of the TE insertion in selected accessions.

Population genetic analysis provides insights into the evolutionary trajectories of phenotypic variation across natural populations. By integrating previously reported phenotypic data on rind background color and striping patterns with SNP data, we performed a GWAS. This analysis identified both the *Mt2* locus and the previously reported *CmAPRR2* locus (Fig. 6A and Fig. S3). Notably, the most significant SNPs were located within the intronic regions of *Mt2* and lie only 131 bp from the TE insertion site. Given that this SNP is absent in both parental lines ‘*S249*’ (or ‘*P114*’) and ‘*13C*’, while the TE insertion is present in both and directly alters the coding region, we propose that the TE insertion is the causal variant underlying *Mt2* (Fig. 6B).

To further examine the association between the TE insertion and the rind mottling phenotype in natural accessions, we leveraged the sequence specificity of the TE insertion breakpoint to perform population-scale genotyping using short-read sequencing data [21] and a previously established TE-genotyping pipeline [25]. Among the 650 genotyped accessions, 493 contained TE while 157 lacked the TE insertion in *CmKNAT2-likes* (Table S5). However, rind pattern phenotypes were only recorded in 250 of these accessions. Statistical analysis revealed that among 182 TE-absent accessions recorded with rind patterns, 68.68% (*n* = 125) exhibited the mottled rind phenotype. In contrast, 72.06% (*n* = 49) of 68 TE-present accessions displayed a nonmottled phenotype, suggesting that TE insertion may suppress mottled formation in natural melon populations (Fig. 6C). To ensure the accuracy of TE genotyping, we performed further validation using polymerase chain reaction (PCR). The TE insertion was found to be significantly associated with the mottled rind phenotype, consistent with our findings (Fig. 6D). These findings suggest that the TE insertion has been subject to widespread selection in natural melon populations and plays a key role in the emergence of the mottled rind trait.

## Discussion

TEs, once considered genomic ‘junk,’ are now recognized as dynamic drivers of crop genome evolution and phenotypic diversity, influencing key agronomic traits through multiple mechanisms, including gene disruption, expression modulation, epigenetic regulation, and genome restructuring. These mobile elements contribute to crop improvement by creating genetic variation that underlies important characteristics such as yield, quality, stress tolerance, and developmental timing, as demonstrated by examples like the wrinkled seed in *Pisum sativum*, which resulted from the insertion of *Ips-r* transposon into *starch branching enzyme 1* (*SBE1*) [26]; the white grape, caused by the insertion of *Gret1* retrotransposon into the promoter of *VvmybA1* [3]; the blood orange, which arose from the insertion of a *Copia*-like retrotransposon adjacent to *Ruby* [27]; and the ‘shooting star’ phenotype in melon, attributed to the recurrent excision of a hAT-like TE in *CmAPRR2* [23]. In the current study, a CACTA-type TIR transposon was identified as the causal variant responsible for the nonmottled rind in ‘*13C*’ and other melon germplasms (Figs 3 and 6). Future research should explore the broader prevalence of TE-mediated trait variation across crops, leveraging advanced genome editing and epigenomic tools to exploit TEs for targeted crop enhancement.

Previous studies showed that the mottled rind in melon is dominant over nonmottled rind, which is controlled by a single gene [17–19].However, recent researches have established that the mottled rind in melon is governed by two dominant loci: *Mt1* and *Mt2* [21, 22, 24]. Of these, *Mt1* has been functionally characterized as *CmAPRR2*, a known regulator of rind pigmentation. In contrast, although *Mt2* has been mapped to the terminal region of chromosome 2 [19, 24], its molecular identity and mechanistic role remain elusive. In the current study, the nonstriped line ‘*13C*’ was crossed with two distinct mottled rind lines, ‘*P114*’ and EMS-induced mutant ‘*S249*’, to generate complementary mapping populations (Figs 1 and 2). However, our analysis of bulked segregant analysis (BSA) using the *DHL92* genome showed no peaks exceeding the confidence interval (Fig. S4), while the linkage signal was successfully identified using the parental line accession (‘*13C*’) genome assembly (Fig. 3C). Therefore, we proposed that two barriers likely prevented *Mt2* isolation: suboptimal reference genome quality and the omission of this transposon variant. Recent studies have demonstrated that a single reference genome is insufficient to capture the full spectrum of sequence diversity within a species. Such a limitation hinders the detection of major structural variants, including copy number variants, presence/absence variants, and translocation variants, which are now recognized as pivotal drivers of genome evolution and key determinants of agronomic traits in crops [12, 15, 25, 28]. Pangenome studies across multiple crop genera have revealed their potential in detecting genetic variations, uncovering functional genes, and facilitating crop genetic enhancement [29–32]. Consequently, initiating pangenome studies for melon crops emerges as a critical research imperative.

Structural variation can have a profound impact on protein function. Interestingly, the ‘*13C*’ mutant of *KNAT2-like2*, resulting from TE insertion, lacks both the ELK domain—essential for nuclear localization—and the Homeodomain, critical for DNA binding. A novel peptide sequence was also introduced (Fig. 4A). Given that nuclear localization is often a prerequisite for transcription factor complex formation, it is plausible that the mislocalization observed in ‘*13C*’ could impair its ability to dimerize. Although we observed both aberrant subcellular localization and reduced dimerization, the causal relationship between these two alterations remains to be elucidated. Further studies—such as forced nuclear localization constructs or domain complementation—may help clarify whether correct nuclear targeting is necessary for dimerization. Exploring this connection would deepen our understanding of *KNOX* gene family regulatory mechanisms.

The study elucidates a TE-driven molecular mechanism for rind mottling. Nevertheless, due to factors such as limited sample size, subtle variations in phenotypic evaluation, and differences in genetic background, 27.94% of the TE+ accessions still exhibited the mottled fruit phenotype. Future investigations into the molecular mechanisms of melon fruit mottling will require larger populations and more accurate phenotypic assessments. Moreover, additional key regulatory genes contributing to fruit mottling remain to be identified. Besides that, genetic inheritance analysis of mottled rind in melon revealed that *Mt1* was epistatic to the *Mt2* locus [22]. Previous study reported that KNOX transcription factors *TKN2* and *TKN4* influence a gradient of fruit chloroplast development through regulation of *SlAPRR2-like* expression in tomato [33]. Given that *Mt2* encodes a KNOX family transcription factor and *Mt1* likely corresponds to *CmAPRR2*, it is reasonable to presume that the epistatic interaction of *Mt1* over *Mt2* may result from *CmKNAT2-like2* functioning upstream of *CmAPRR2* to regulate its expression either directly or indirectly. Interestingly, a recent study on watermelon rind coloration demonstrated that a KNAT2-like2 homolog can directly regulate *CsAPRR2* [34]. Nevertheless, the precise molecular interplay between *Mt2* and *Mt1* remains to be fully deciphered in the following study.

## Materials and methods

### Plant materials and growth conditions

The cultivated *agrestis* accession ‘*13C*’—characterized by a light green rind at the immature stage and light gray at maturity—and wild African accession ‘*P114*’ (mottled rind)—exhibiting a mottled rind pattern—were used to develop NILs differing in rind appearance (mottled vs nonmottled). The NILs served as the parental lines for generating an F_2_ segregating population. Additionally, an EMS-mutagenized population was created using ‘*13C*’ as the parent. From this population, a dominant mottled-rind mutant, designated ‘*S249*’, was identified in the M_1_ population. The F_1_ generation was produced by crossing ‘*13C*’ and ‘*S249*’, and the corresponding F_2_ population was derived from self-pollinated F_1_ individuals. These plants were employed for phenotypic characterization, map-based cloning, and RNA-seq. All experiments were conducted in a plastic greenhouse located in the Agricultural Hi-tech Industry Zone, Jimo, Shandong, China (36°56′N, 120°21′E) during the growing seasons from 2021 to 2025.

### Mapping strategy and sequence analysis

A traditional map-based cloning approach was employed to identify the *Mt2* locus using segregating populations derived from the NILs. SNPs and InDel variants within the chromosomal region associated with the mottled rind phenotype were converted into molecular markers for genotyping.

To map *Mt2* in the dominant mottled mutant ‘*S249*’, we performed MutMap^+^ analysis [35]. Genomic DNA was extracted from young leaves of 20 mottled and 20 nonmottled individuals using the CTAB method [36]. Paired-end sequencing libraries (150-bp read length, ~350-bp insert size) were constructed and subjected to whole genome resequencing on the Illumina NovaSeq 6000 platform, yielding approximately 30× genome coverage per bulk. BSA was initially performed using the published DHL92 (V4) reference genome. However, this approach failed to identify significant loci associated with the mottled rind trait (Fig. S4). To minimize background variation and improve mapping resolution, a refined MutMap^+^ analysis was carried out using our team’s high-quality genome assembly of ‘*13C*’ as the reference. Clean reads were aligned to the ‘*13C*’ genome using BWA (0.7.17-r1188) with default parameters [37]. SAMtools (v1.11) [38] was then used for file conversion (SAM to BAM), sorting, duplicate removal, and indexing. SNPs and InDels were identified using the Genome Analysis Toolkit (4.1.4.0) [39], followed by functional annotation with SnpEff (v4.3) [40]. To enhance mutation specificity, further SNP filtering was conducted based on two criteria: (i) retention of EMS-typical transitions (G → A or C → T) and (ii) selection of SNPs with an SNP index ≤0.8 in the dominant (wild-type) pool and ≥0.2 in the mottled (mutant) pool.

A ΔSNP index plot was generated using a custom R script designed to analyze individual SNPs across the genome. For each sliding window, the x-axis position of the averaged SNP index corresponded to the midpoint between the first and fifth SNPs. Variants showing a ΔSNP index greater than 0.6 and supported by a read depth exceeding 10 were considered high-confidence candidates. These SNPs were subsequently converted into cleaved amplified polymorphic sequence (CAPS) markers using the dCAPS Finder 2.0 tool (http://biology4.wustl.edu/dcaps/). All primers were synthesized by Sangon Biotech, and their sequences are listed in Table S1. Recombinant individuals within the F₂ population were genotyped using the polymorphic CAPS markers for fine mapping.

### Transmission electron microscope

Fruit rind tissues (~0.5 cm^2^) were collected from ‘*13C*’ and ‘*S249*’ at 21 days postanthesis. Samples were fixed in 2.5% glutaraldehyde for 12 hours followed by three washes in 1× PBS buffer. Dehydration was carried out through a graded ethanol series. The dehydrated samples were then dried, sputter coated with gold, and imaged using a Hitachi HT7800 (Hitachi, Japan).

### Phylogenetic analysis

Multiple sequence alignments were performed using the MUSCLE algorithm with default parameters [41]. The resulting alignments were used to construct phylogenetic trees in MEGA11 (https://megasoftware.net/) using the neighbor-joining method [42]. Tree visualization and annotation were subsequently refined using the Interactive Tree of Life (iTOL) platform (https://itol.embl.de/).

### Stable genetic transformation in melon

To achieve CRISPR/Cas9-mediated genome editing of *CmKNAT2-like2*, specific sgRNA target sites were designed using the CRISPR-GE online tool (http://skl.scau.edu.cn/home/). A PCR fragment containing the sgRNA cassette was amplified using the pCBC-DT1T2 vector as a template and subsequently cloned into the binary vector pBSE402 via the In-Fusion cloning method [43, 44]. Genetic transformation of melon (‘*P147*’) was performed following a previously established protocol [45].

### RNA-seq analysis

Peel samples at 21 days postanthesis were collected from two groups: ‘*13C*’ vs ‘*S249*’, and mottled vs nonmottled regions of ‘*P114*’. Total RNA was extracted and subjected to transcriptome sequencing using the BGI T7 platform. Each group included three biological replicates. Clean reads from all six samples were aligned to the ‘*13C*’ reference genome using HISAT2 (v2.2.1) with default parameters [46]. Gene expression levels were quantified in FPKM (fragments per kilobase of transcript per million mapped reads) using StringTie (v2.1.5) [47]. DEGs between the two conditions were identified based on an adjusted *P* value <0.05 and |log_2_ (fold change)| ≥ 1. Gene ontology enrichment analysis was performed using the ‘clusterProfiler’ package in R.

### Genome-wide association study for melon mottled rind

The genome-wide association analysis of the mottled rind trait was conducted using publicly available phenotypic and SNP datasets [15, 48]. A significance threshold of *P* ≈ 2.63 × 10^−6^ was used to identify loci associated with the trait. To further explore the genomic region surrounding *Mt2*, linkage disequilibrium (LD) patterns were analyzed using LDBlockShow (https://github.com/BGI-shenzhen/LDBlockShow).

### Transposon insertion genotyping of *Mt2* using short-read sequencing data

Given the repetitive nature of TE, the specificity of the *Mt2*-associated TE and its flanking sequences was first evaluated through BLAST alignment. This analysis confirmed that the 200-bp region flanking the TE insertion site is unique within the ‘*13C*’ genome. Following previously established methodologies for TE genotyping [25] using short-read sequencing data [21], the presence of the TE was determined by identifying reads spanning the insertion breakpoint. Samples with two or more breakpoint-spanning reads were classified as containing the TE, while those with one or no such reads were considered TE absent. To assess the reliability of this approach, PCR-based validation was performed on a subset of 25 samples.

### Subcellular localization

The coding sequences of *CmKNAT2-like2* from both ‘*S249*’ and ‘*13C*’ were individually cloned into the p1305.4-GFP vector (primer sequences are provided in Table S1). These constructs were transiently expressed in *Nicotiana benthamiana* leaf epidermal cells via *Agrobacterium tumefaciens* strain GV3101-mediated infiltration. Confocal fluorescence imaging was conducted 36–48 hours postinfiltration using a HOOKE S3000 spinning disk confocal microscope. GFP fluorescence was excited at 488 nm, and images were captured and subsequently processed with ImageJ software.

### Y2H assays

Y2H assays were conducted using the Y2HGold-GAL4 system (Coolaber) and the yeast strain Y2HGold, following the manufacturer’s instructions. The coding sequences of *CmKNAT2-like2* from ‘*S249*’ and ‘*13C*’ were cloned into the pGADT7 and pGBKT7 vectors, generating four constructs: pGADT7-CmKNAT2-like2*^S249^*, pGADT7-CmKNAT2-like2*^13C^*, pGBKT7-CmKNAT2-like2*^S249^*, and pGBKT7-CmKNAT2-like2*^13C^* (primer sequences are listed in Table S1). Yeast transformations were performed according to the combinations illustrated in Fig. 4. Aliquots (2 μl) of yeast cultures (OD_600_=0.2) expressing specific constructs were plated on selective media: SD-Trp-Leu-His-Ade, SD-Trp-Leu-His, and SD-Trp-Leu. Protein–protein interactions were inferred by comparing the growth of experimental groups to the corresponding empty AD vector controls.

### Pull-down assay

The coding sequence of *CmKNAT2-like2^S249^* was cloned into the pGEX-4T-1 vector and expressed in *Escherichia coli* BL21 (DE3) cells to produce the GST-CmKNAT2*^S249^* fusion protein. Similarly, recombinant constructs encoding CmKNAT2-like2*^S249^* (pET22B-NEXT) and CmKNAT2-like2*^13C^* were expressed in BL21 (DE3) to produce His-tagged fusion proteins. Primers are listed in Table S1. For GST pull-down analysis, 500 μg of purified GST-CmKNAT2-like2*^S249^* was incubated with an equimolar amount (500 μg) of His-tagged protein in the presence of glutathione-agarose beads at 25°C for 4 hours. Following incubation, beads were washed six times to remove unbound components, then boiled in 1× SDS sample buffer to elute bound proteins. Samples were separated and analyzed by western blot using corresponding antibodies.

## Supplementary Material

Web_Material_uhaf195

## Data Availability

Relevant data can be found within the paper and its supporting materials. The sequencing data that support the findings of the Mutmap^+^ analysis and RNA-seq analysis have been deposited in the Sequence Read Archive under accession number PRJNA1284442. Other data of this study are available from https://zhanglab.qau.edu.cn/melow/index.php.
