# Supplementary material for: A transposon insertion in CmKNAT2-like2 disrupts mottled rind formation in melon (Cucumis melo L.)
Source: Hortic Res. 2025 Jul 28;12(10):uhaf195. doi: 10.1093/hr/uhaf195 (PMC12549080; doi:10.1093/hr/uhaf195)
Supplement: Web_Material_uhaf195 [file web_material_uhaf195.zip › Supplementary Figure.docx]

**A transposon insertion in *CmKNAT2-like2* disrupts mottled rind formation in melon (*Cucumis melo* L.)**

**Shuai Li^2,3†^, Jing Feng^1†^, Xinxiu Chen^1†^, Yuanchao Xu^3^, Yuhao Song^1^, Fanfan Chen^1^, Yang Li^1^, Naonao Wang^1^, Jianlei Sun^2^, Zhonghua Zhang^1^, Sen Chai^1*^**

**Supporting Information**

**Supplementary Figure 1.** The CmKNAT2-like2*^13C^* protein exhibits reduced nuclear localization capability.

**Supplementary Figure 2.** Fruit of ‘*13C*’ and ‘*S249*’ at different developmental stages. Scale bars, 2 cm.

**Supplementary Figure 3.** GWAS analysis of fruit rind mottling in melon.

**Supplementary Figure 4.** BSA analysis using the DHL92 (v4) reference genome.


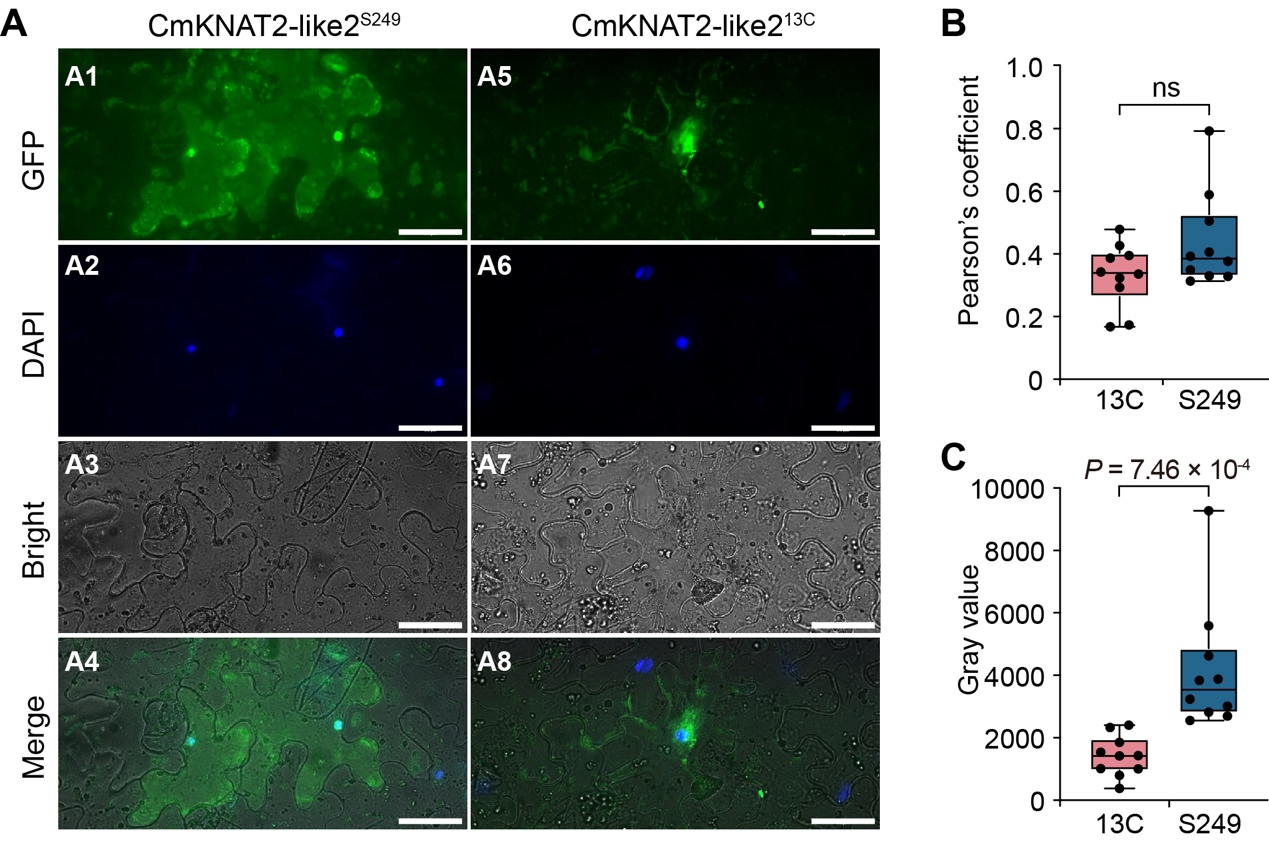


**Supplementary Figure 1.** The CmKNAT2-like2*^13C^* protein exhibits reduced nuclear localization capability. (A) Subcellular localization of CmKNAT2-like2*^S249^* (A1-A4) and CmKNAT2-like2*^13C^* (A5-A8). Bars, 50 μm. (B) Statistical results of the colocalization of CmKNAT2-like2*^S249^* and CmKNAT2-like2*^13C^*. Data are presented as mean ± SD (n=10, *t*-test). (C) Gray value analysis to indicated the fluorescence intensity of the CmKNAT2-like2*^S249^* and CmKNAT2-like2*^13C^* in nuclear localization. Data are presented as mean ± SD (n=10, *t*-test).


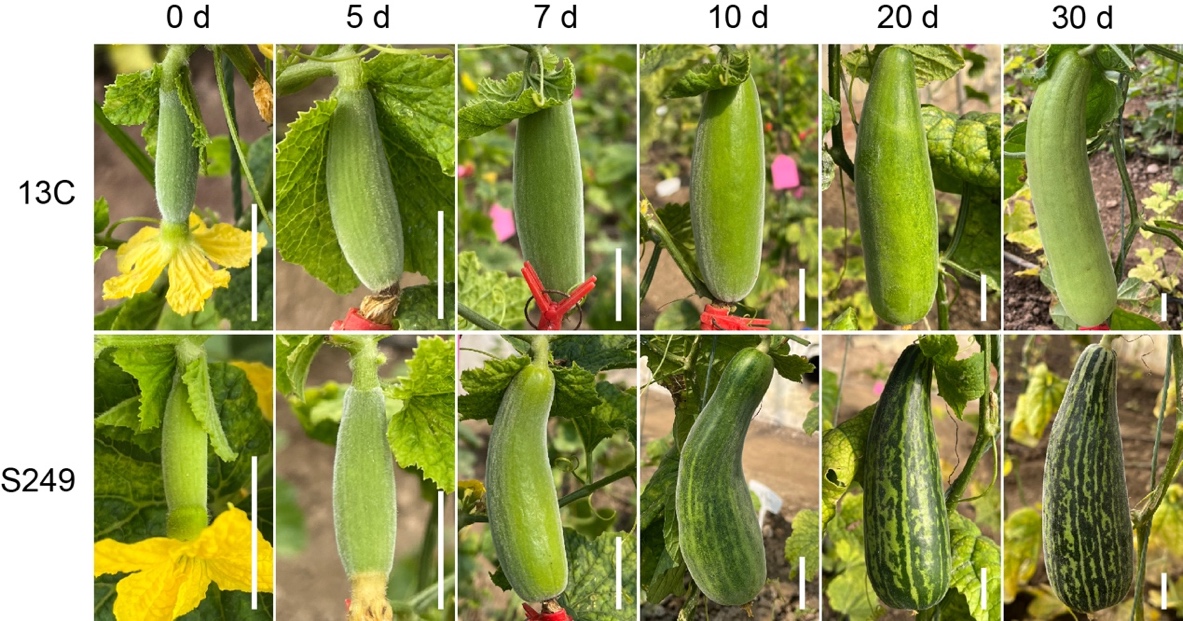


**Supplementary Figure 2.** Fruit of ‘*13C*’ and ‘*S249*’ at different developmental stages. Scale bars, 2 cm.


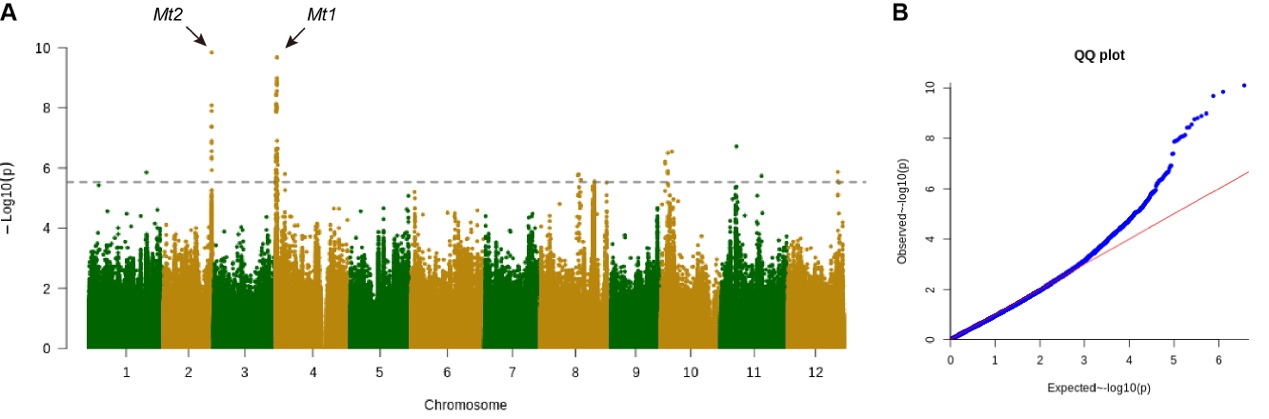


**Supplementary Figure 3.** GWAS analysis of fruit rind mottling in melon. Manhattan plots (A) and quantile-quantile plots (B) for GWAS on fruit rind mottling.


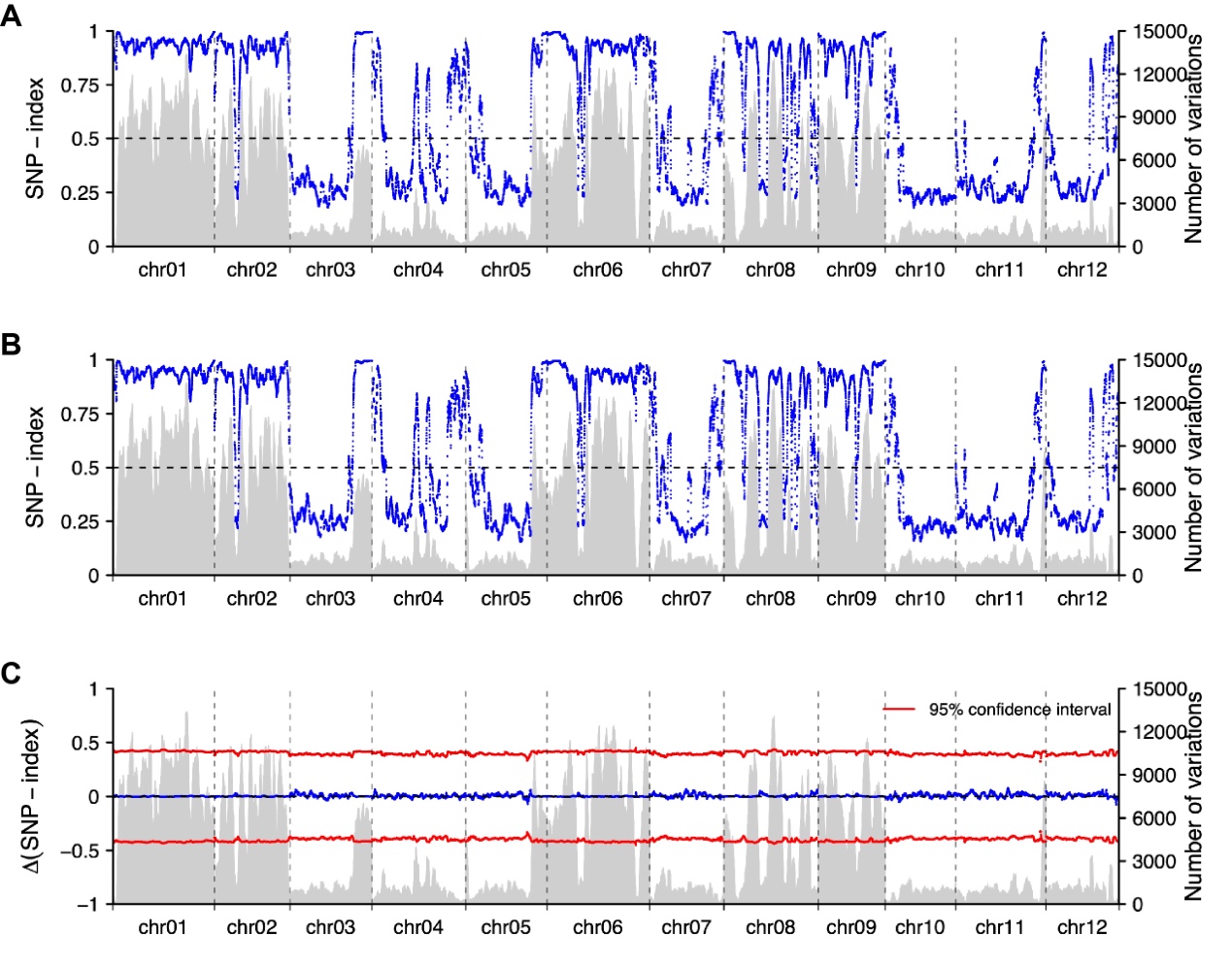


**Supplementary Figure 4.** BSA analysis using the DHL92 (v4) reference genome. (A) SNP-index of M-pool. (B) SNP-index of N-pool. (C) ΔSNP-index between M-pool and N-pool.
